# Development of body knowledge as measured by arm differentiation in infants: From global to local?

**DOI:** 10.1111/bjdp.12309

**Published:** 2019-11-09

**Authors:** Lisa Jacquey, Sergiu Tcaci Popescu, Judith Vergne, Jacqueline Fagard, Rana Esseily, Kevin O’Regan

**Affiliations:** ^1^ Integrative Neuroscience and Cognition Center CNRS Université Paris Descartes France; ^2^ Laboratoire Ethologie Cognition Développement Université Paris Nanterre France

**Keywords:** body knowledge, infants, sensorimotor contingencies

## Abstract

The ability to sense and use the body parts in an organized and differentiated manner is a precursor of body knowledge in infancy. To acquire this ability, the infant's brain might explore the perceptual consequences of its bodily actions. Undifferentiated body movements would gradually be replaced by more precise actions. Only a very few studies have tested this ‘global‐to‐local’ hypothesis, and none of them have so far been replicated. In this study, we assessed arm differentiation in 4‐, 6‐, and 8‐month‐old infants using a new contingency detection task in which infants have to detect a contingency between one of their arms’ activity and an audiovisual stimulus on a screen. We found that 4‐ to 8‐month‐old infants seem to be able to use their arms in a differentiated manner. However, surprisingly, we were not able to show a developmental trend in arm differentiation between 4 and 8 months of age.

**Statement of contribution**

***What is already known on this subject?***

- Foetuses and infants possess coarse control of their body and may be sensitive to sensory feedback caused by their own movements.
- Body knowledge might develop during the first year of life in what can be called a ‘global‐to‐local’ manner. Nevertheless, the precise age at which infants come to possess well‐differentiated local body knowledge requires further investigation.

***What the present study adds?***

- 4‐ to 8‐month‐old infants seem able to use their arms in a differentiated manner when exposed to an audiovisual stimulation contingent on movements of one of their arms.
- However, we found no developmental trend in arm differentiation between 4 and 8 months of age.
- We hypothesize that infants' sensitivity to sensorimotor contingencies and their ability to narrow down contingencies to a specific limb might evolve with age as a function of the infant's current sensorimotor interests.

## Background

During the first years of life, infants and toddlers progressively acquire the ability to use their body to interact efficiently with their physical and social environment (Piaget, 1936/1952; Rochat & Goubet, 2000). While the importance of this ability in development cannot be disputed, its study is limited by certain difficulties. One problem is that authors have defined this ability in different ways, resulting in a multitude of overlapping concepts of body knowledge, such as body schema, body image(s), visuospatial body map, and body semantics (see de Vignemont, 2010 for a review). Another problem is that often such notions refer to the concept of ‘mental representation’, whereas it is not clear what is meant by this nor how to demonstrate its existence in infants. For these reasons, we prefer in the present paper to coin a new term, namely ‘body know‐how’, that we intend to be restricted to practical aspects of body knowledge that may not involve internal representations and that may be constituted by a collection of skills. More precisely, we define ‘body know‐how’ as the ability to sense and use the body parts in an organized and differentiated manner. In the present article, we examine the hypothesis that body know‐how develops from a global state where infants have fairly undifferentiated knowledge of their bodies, to a better localized form of know‐how that allows infants to use their limbs in a differentiated manner. We investigated this hypothesis by assessing arm differentiation in 4‐, 6‐, and 8‐month‐old infants using a new contingency detection task in which infants have to detect a contingency between one of their arms’ activity and an audiovisual stimulus on a screen. Before presenting our experiment, we start by reviewing studies investigating how during the foetal stage and early infancy sensitivity to sensorimotor and sensory–sensory contingencies supports the early development of body know‐how. We then detail the few studies that more precisely document body know‐how development in the first months of life.

### Body know‐how in early infancy

We hypothesize that the development of body know‐how is rooted in infants’ sensitivity to the consequences of their actions (i.e., sensorimotor contingencies) and to the correspondence between events in different sensory modalities (i.e., sensory–sensory contingencies). Sensitivity to sensorimotor contingencies seems already to be present during the last 3 months of pregnancy and at birth, since foetuses and newborns seem implicitly aware of the consequences of some of their actions. For example, foetuses may open their mouths in anticipation when their hands approach their face (Myowa‐Yamakoshi & Takeshita, 2006; see also Fagard, Esseily, Jacquey, O’Regan, & Somogyi, 2018; Reissland & Austen, 2018 for reviews) and 4‐week‐old infants distinguish their own spontaneous touch of their cheeks with one hand (actively self‐touching) from when an experimenter touches their cheeks (external touch) (Rochat & Hespos, 1997). Around 2 months of age infants seem to become able to modulate sucking when this generates sensory changes (Rochat & Striano, 1999).

Sensitivity to the correspondence between events in different sensory modalities might also play a role in the early development of body know‐how. Indeed, infants’ sensitivity to the correspondence between visual and tactile inputs of stimuli applied on their body seems already present at birth (Filippetti, Johnson, Lloyd‐Fox, Dragovic, & Farroni, 2013; Zmyj, Jank, Schütz‐Bosbach, & Daum, 2011). Filippetti *et al. *(2013) showed 1‐day‐old newborns videos of upright and inverted infant faces being touched on their cheeks either in synchrony or out of synchrony with actual stroking felt on the newborn's own face. The authors showed that the newborns preferred to look at synchronous visuotactile stimulation rather than asynchronous stimulation, but only in the upright face condition. The visuotactile integration observed in Filippetti's study has also been found for other body parts (legs) in 7‐ and 10‐month‐old infants (Zmyj *et al.*, 2011). Moreover, infants’ sensitivity to the correspondence between visual and proprioceptive feedback from their body movements seems to appear around 2–3 months of life. For example, from 3 months infants are able to discriminate contingent visual feedback caused by their body movements from (temporally or spatially) non‐contingent visual feedback (Bahrick & Watson, 1985; Rochat & Morgan, 1995).

In sum, these studies demonstrate that foetuses and very young infants possess coarse control of their body and may be sensitive to contingent feedback from their own movements and to the correspondence between events in different sensory modalities. However, these studies do not inform us about the degree to which infants know the precise structure of their bodies, in particular whether they use their body parts in a differentiated manner. Two types of approach have been used to answer this question: infants’ responses to tactile stimuli and infants’ sensitivity to sensorimotor contingencies.

#### Limb‐differentiating responses to tactile stimuli

A first approach has been to measure infants’ neural responses to tactile stimulation applied on different body parts. Thus, Meltzoff, Saby, and Marshall (2018) and Saby, Meltzoff, and Marshall (2015) observed that from 2 months of age, evoked potential responses to touch stimulations on the mouth, hands and feet were organized somatotopically in a way similar to that found in adult brains. But to what extent does this neural organization have a behavioural correspondence? This has been studied via infants’ motor responses to vibrotactile stimulation applied to different areas of the body. Thus, Somogyi *et al. *(2018) showed that infants’ motor responses to vibrotactile stimulation become progressively organized in a topographical manner during the first months of life. In a longitudinal study from 3 to 6 months of age, these authors stimulated infants with a vibrating buzzer applied to one hand or foot. They found that at 3 months infants responded with global movements of their body and, at 5–6 months, infants responded more specifically with the hand or foot stimulated by the buzzer. Other studies showed that already at the earliest ages tested (6 months for the hands and 4 months for the feet) infants can locate an unseen vibrotactile stimulus on the hands (Bremner, Mareschal, Lloyd‐Fox, & Spence, 2008) and on the feet (Begum Ali, Bremner & Spence, 2015). In these studies, infants showed more manual and visual orientation towards the stimulated limb compared with the non‐stimulated limb. In summary, these studies tell us that at the neural level, infants’ body know‐how seems to be established from at least 2 months of age, since brain imagery in infants provides evidence of a topographical organization of the body. However, on the behavioural side, current data indicate that body know‐how becomes localized only around 4–6 months, suggesting that limb‐differentiating responses at the behavioural level are established later than at the neural level. Nevertheless, this statement needs to be taken with caution, given that Bremner's team (Begum Ali et al., 2015; Bremner *et al.*, 2008) showed evidence for local body know‐how at the youngest age tested (4 months of age) and that younger infants have not yet been tested with the same protocol.

#### Limb‐differentiating sensitivity to sensorimotor contingencies

Around 3–4 months, infants are already able to produce task‐specific actions with their limbs (for example knee flexion or extension) when these actions generate movements of a mobile above them (Angulo‐Kinzler, Ulrich, & Thelen, 2002; Sargent, Schweighofer, Kubo, & Fetters, 2014; Thelen, 1994). But to what extent can infants specifically move one limb when only movements of this limb generate a contingent effect? Limb‐differentiating sensitivity to sensorimotor contingencies was tested in very young infants by van der Meer (1997) and van der Meer, van der Weel, and Lee (1995, 1996) who showed that in some conditions, even 2‐week‐old infants can specifically move one arm in order to bring it into sight. In older infants, limb‐differentiating sensitivity to sensorimotor contingencies has mainly been investigated using the ‘mobile’ paradigm. In this paradigm, one of the infant's limbs is attached to a mobile hanging over the infant's head in such a way that moving the limb makes the mobile move in a contingent manner (Rovee & Rovee, 1969). Using this method, it has been shown that 3‐ to 4‐month‐old infants can move one limb specifically when movements of this limb activate the mobile (Angulo‐Kinzler, 2001; Heathcock, Bhat, Lobo, & Galloway, 2005; Rovee‐Collier, Morrongiello, Aron, & Kupersmidt, 1978; Watanabe, Homae, & Taga, 2011; Watanabe & Taga, 2006, 2009). More precisely, Watanabe and Taga (2006) found a developmental trend like that observed in Somogyi *et al. *(2018): when one arm was connected to the mobile, over the course of the experiment, 2‐month‐old infants increased the activity of their four limbs, 3‐month‐old infants increased the activity of their arms but not of their legs, and 4‐month‐old‐infants increased the activity of the connected arm only. However, this ability of 3‐ to 4‐month‐old infants to use their limbs in a differentiated manner has not been replicated in other studies using the same paradigm: in Thelen (1994), the authors tested 3‐month‐old infants and found a difference between the connected and unconnected leg movements but only in velocity and not in frequency, and in Angulo‐Kinzler *et al. *(2002), the authors did not find any difference between the connected and unconnected leg movements in 3‐month‐old infants. Thus, the existing literature does not allow us to conclude with confidence whether infants possess local body know‐how and are able to use their limbs in a differentiated way from the age of 3–4 months, or whether this capacity only emerges later. In addition, a real understanding of the development of body know‐how in young infancy would require data from infants over 4 months of age tested in sensorimotor contingency tasks, which is not available to our knowledge (probably because the mobile paradigm is not adapted to older babies – cf. Hartshorn & Rovee‐Collier, 1997).

To sum up, this literature review suggests that body know‐how develops in what might be called a ‘global‐to‐local’ manner, that is from a state in which infants use their whole body in an undifferentiated way to a differentiated state in which infants are able to use their limbs independently of each other in an adapted way. Indeed, the studies mentioned above show that at first infants move their whole bodies and that later they are able to move one specific limb in response to a stimulation (Somogyi *et al.*, 2018) or when movements of this limb produce movements of a mobile above them (Watanabe & Taga, 2006). Nevertheless, the precise age at which infants come to possess well‐established local body know‐how requires further investigation. In particular, there is a lack of studies assessing body know‐how in infants older than 4 months of age. In the current study, we will attempt to fill this gap by studying the development of body know‐how between 4 and 8 months of age by exploiting infants' sensitivity to sensorimotor contingencies.

### The present study

In the present study, we exposed 4‐, 6‐, and 8‐month‐old infants to a real‐time contingency between movements of one of their arms and an audiovisual stimulation displayed on a screen. An age‐matched control group saw an equally salient non‐contingent audiovisual stimulation. We expected first to find a difference in activity between the infants in the contingent and non‐contingent groups. We expected that this difference might consist in greater activity and/or greater increase in activity over the course of the session in the contingent group than in the non‐contingent group and that the difference between groups would increase with age. This difference in motor activity between the two groups is the behavioural measure we adopted as a sign of sensitivity to the sensorimotor contingency in infants in the contingent group. Our first purpose was thus to check whether 4‐, 6‐, and 8‐month‐old infants were sensitive to the contingency we had established. The second purpose was to assess whether they would be able to use their arms in a differentiated manner, that is to restrict their movements to the particular arm that controlled the contingency. We expected that with age, infants would progressively become more able to use the connected arm in a differentiated manner, that is that they would show greater activity and/or a greater increase in activity over the course of the session only in the connected arm.

## Method

### Participants

The participants were thirty‐four 4‐month‐old infants (mean age = 125 days, *SD* = 6 days, range = 113–137 days), thirty‐five 6‐month‐old infants (mean age = 184 days, *SD* = 8 days, range = 167–196 days), and thirty‐five 8‐month‐old infants (mean age = 243 days, *SD* = 9 days, range = 226–259 days) (see Table 1 for details). Infants were recruited from a list of interested local middle‐ to upper‐middle class families. Each family gave their written informed consent. The experimental protocol was approved by the University Paris Descartes ethics committee. Infants were assigned to the contingent or non‐contingent condition as they became available until a count of at least 16 infants per age and condition was reached. This number was chosen based on numbers used in similar paradigms (10 infants in Heathcock *et al.*, 2005; 10 infants in Rovee‐Collier *et al.*, 1978; 16 infants in Watanabe & Taga, 2006). Twenty additional infants were tested but had to be excluded due to fussiness (*N* = 13), premature birth (*N* = 2), or technical problems (*N* = 5) (see Table 1 for details).

**Table 1:** Information on participants by group (contingent or non‐contingent) and age (4, 6, or 8 months of age)

	Contingent group		Non‐contingent group	
	Included	Excluded	Included	Excluded
4 months	17 infants 9 girls and 8 boys mean age = 121 days *SD* = 4 days range = (113–130)	2 infants Fussiness (*N* = 2)	17 infants 9 girls and 8 boys mean age = 128 days *SD* = 6 days range = (119–137)	0 infant
6 months	18 infants 7 girls and 11 boys mean age = 186 days *SD* = 8 days range = (168–196)	2 infants Fussiness (*N* = 1) Technical (*N* = 1)	17 infants 3 girls and 14 boys mean age = 182 days *SD* = 9 days range = (167–195)	6 infants Fussiness (*N* = 3) Prematurity (*N* = 1) Technical (*N* = 2)
8 months	17 infants 11 girls and 6 boys mean age = 240 days *SD* = 11 days range = (226–257)	8 infants Fussiness (*N* = 5) Prematurity (*N* = 1) Technical (*N* = 2)	18 infants 7 girls and 11 boys mean age = 246 days *SD* = 6 days range = (236–259)	2 infants Fussiness (*N* = 2)

### Experimental set‐up

**Figure 1:**
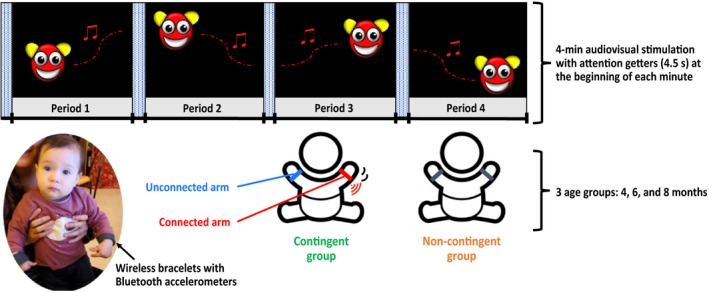
We exposed 4‐, 6‐, and 8‐month‐old infants to a real‐time contingency between movements of one of their arms (connected arm) and an audiovisual stimulation displayed on a screen. The side (right or left) of the connected arm was counterbalanced between infants. Arm movements were measured at 50 Hz by Bluetooth‐connected accelerometers worn on the baby's wrists. The experiment lasted 4 min separated into four periods of 55.5 s each. Before the beginning of each period, an attention‐getter of duration 4.5 s occurred, consisting of an expanding white disc displayed on the screen, accompanied by a metallic sound. Age‐matched control groups were provided with an equally salient non‐contingent audiovisual stimulation. We compared: (1) arm activity in the contingent group versus the non‐contingent group and (2) for the contingent group alone, arm activity of the connected arm versus the unconnected arm.

The experimental booth, constructed with ceiling‐to‐floor black curtains, contained a table covered with black fabric and a chair in front of which were placed a 23‐inch computer screen and two loudspeakers placed symmetrically on either side of the screen. Two video cameras filmed the infant from the front and above. During the experiment, infants were seated on a parent's lap in front of the screen at approximately 60 cm. On each arm, infants wore a custom‐made bracelet containing an accelerometer (MetaWear RG, MbientLab, San Francisco, CA, USA) communicating via low‐energy Bluetooth 4.0, ASUS, Taipei, Taiwan, Republic of China with a computer (Figure 1).

### Measure of instantaneous arm activity

The accelerometers sampled the acceleration of each arm, measured in units of g (the earth's gravitational acceleration) in the *x*, *y*, and *z* directions at a frequency of 50 Hz. The *instantaneous acceleration* at time *t* for each arm was calculated as the square root of the sum of the squares of the *x*, *y*, and *z* values. This value was then low‐pass filtered by computing: value(*t*) = 0.015 × value(*t* − 1) + 0.985 × instantaneous acceleration(*t*). The weights 0.015 and 0.985 were chosen during a pilot study (32 infants tested) in order to reject peak values. We considered that this low‐pass filtered acceleration value represented a measure of each arm's ‘instantaneous activity’.

### Contingent audiovisual stimulation

We used the ‘instantaneous activity’ value to control an audiovisual stimulus so that it changed position on the screen in real time depending on movements of one of the infant's arms (the *connected* arm) and was independent on the movements of the other arm (the *unconnected* arm). The contingent audiovisual stimulus (Figure 1) consisted of a highly salient red‐and‐yellow smiley on a black background accompanied by a 2 s 20 dB bell‐sound obtained from an open‐access sound bank. The smiley was continually visible on the screen, and its displacement was a function of the current ‘instantaneous activity’ level as defined above. In this way, our contingency was similar to what would happen if our smiley was a real object moved by the force exerted on it by the connected arm. More precisely, the smiley's motion was continuously subject to a ‘force’ calculated from the activity of the connected arm and to a ‘friction’ dependent on the displacement of the smiley itself. We used the equation: next displacement (in pixels) = force coeff × instantaneous activity − friction coeff × previous displacement. The coefficients of the force (.004) and the friction (.001) components were determined during pilot trials and were kept the same for all infants. The direction of motion was not determined by the direction of arm motion, but changed randomly in a way so as to keep the smiley continually on the screen. The auditory contingency that accompanied the visual contingency consisted in a bell that sounded once every time the speed of the smiley on the screen passed a threshold value and was only played again when the smiley had stopped moving and was then re‐activated by the threshold being again passed. The threshold (2 pixels/20 ms) was determined during pilot trials and was kept the same for all infants.

### Design and procedure

#### Design

For each of the three age groups (4, 6, and 8 months), infants were randomly assigned to the *contingent* (experimental) group or the *non‐contingent* (control) group, making a total of six groups. Infants in the contingent group were exposed to contingent audiovisual stimulation generated by movements of their connected arm (Figure 1). The side (right or left) of the connected arm was counterbalanced between infants. Infants in the non‐contingent group were exposed to a comparable but non‐contingent audiovisual stimulation. This non‐contingent stimulation was specific to each age group and was made by taking the stimulation created by one of the infants of the same age in the contingent group. Indeed, in the non‐contingent condition, the amount of movement of the smiley and of sounds increased over time and corresponded to the expected outcome in the contingent condition. This ensured the same amount of arousal in both conditions.

#### Procedure

Infants were seated on their parents’ lap in front of the screen and exposed to the contingent or non‐contingent audiovisual stimulation for 4 min. We opted for a 4‐min experiment because, on the basis of a pilot study, we considered this duration as an optimal balance between a sufficient contingency exposure and a time period that was short enough not to exclude too many babies because of fussiness. In order to maintain the infant's attention, an attention‐getter consisting of a bright expanding white disc accompanied by a jangling keys sound was displayed at the centre of the screen for 4.5 s before the beginning of each period. The experiment was thus divided into four periods of 55.5 s that we analysed separately in order to evaluate the evolution of the infant's behaviour over the course of the experiment. Parents were instructed to hold their infant at the waist so that both her or his arms were free and to maintain the infant seated as much as possible. Parents were also instructed not to interact with their infant and to look down away from the screen during the experiment.

### Data processing

#### Coding of looking time

The videos were analysed frame by frame using *Psycode* (http://psy.ck.sissa.it/PsyCode/PsyCode.html) to ensure that infants were attentive to the experiment in each group. A second observer coded 30% of the infants’ videos offline. The percentage agreement on infants’ looking times between the two observers averaged 95%.

#### Arm activity

We averaged the instantaneous activity of each arm (see definition above) and the mean instantaneous activity of both arms (we will call this the ‘combined arm activity’) over each of the four periods of the experiment (55.5 s). For the contingency sensitivity assessment, we based our analysis on ‘combined arm activity’ because in the non‐contingent group there was no connected or unconnected arm, and for the assessment of arm differentiation, we based our analysis separately on the connected arm and unconnected arm activities.

## Results

### Looking time analysis

The looking time analysis confirmed that there was no difference in looking time across contingent and non‐contingent groups, *F*(1, 103) = 0.076, *p* = .783, and no difference across age groups, *F*(2, 102) = 2.119, *p* = .126, or interaction, *F*(2, 102) = 1.466, *p* = .236. It should be noted here that the time and direction of infants' gaze could also have been used as indicators of infants' sensitivity to contingency and/or of their ability to use their arms in a differentiated manner. Nevertheless, without eye‐tracking data, it was impossible to accurately determine the direction of the gaze from our video records. We did however note that during the experiments infants very rarely looked at their arms, whether in the contingent or non‐contingent group, which seems to be confirmed by the small proportions of off‐screen looking times in each group (23% in the contingent group and 25% in the non‐contingent group).

### Main results

#### Contingency sensitivity assessment

**Figure 2:**
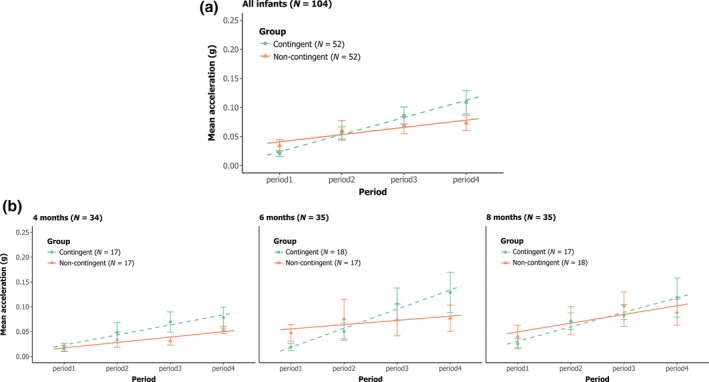
Means and standard errors of the mean of the combined arm activity (calculated as the mean of both arms’ activity) over the four periods of the experiment and the corresponding regression lines for the contingent group (green discs and dashed lines) and the non‐contingent group (orange triangles and solid lines). (a) all infants (b) separated by age (4, 6, and 8 months).

Figure 2 presents results of infants’ combined arm activity over the four periods of the experiment for each group for all infants (Figure 2a) and at each age (Figure 2b). We expected that infants in the contingent group would show higher combined arm activity (calculated as the mean of both arms’ activity) or higher increase in combined arm activity over the experiment compared with infants in the non‐contingent group. We also expected that this difference in arm activity between infants in the contingent and the non‐contingent group would gradually increase with age (4, 6, and 8 months of age).

We see in the top graph with all infants (Figure 2a) that combined arm activity increases over the course of the experiment in both groups and that the rate of increase in combined arm activity over the course of the experiment is higher in the contingent group as compared to the non‐contingent group. This is confirmed in a repeated measures ANOVA by a significant main effect of period, *F*(2.139, 209.625) = 19.029, *p* « .0001, η^2^p = .163, and a significant interaction between period and group, *F*(2.139, 209.625) = 3.107, *p* = .043, η^2^p = .031. There is no significant main effect of group, *F*(1, 98) = 0.288, *p* = .593. In order to better understand the significant interaction between period and group, we performed linear regressions for the combined arm activity of each infant over the four periods of the experiment and calculated the mean of the slopes of the regressions for each group (contingent and non‐contingent). Using a one‐tailed *t*‐test, we tested the hypothesis that the means in the contingent group would be higher than in the non‐contingent group. We found a significant difference in slopes of combined arm activity between the contingent and the non‐contingent groups, *t*(102) = 1.123, *p* = .013. The mean of the slopes of combined arm activity in the contingent group was 0.3 (*SEM* = 0.046), meaning that the combined arm activity's mean in this group increased by 30% at each period of the experiment, going from 0.021 to 0.109 between the first and the last period. In the non‐contingent group, the mean of the slopes of combined arm activity was 0.1 (*SEM* = 0.031), which means that the mean combined arm activity in this group increased by 10% at each period of the experiment, going from 0.035 to 0.073 between the first and the last period.

The lower graphs (Figure 2b) show the results separately for the three age groups. The slopes are slightly different between age groups, but this difference is not significant, as the ANOVA shows no effect of age, *F*(2, 98) = 1.533, *p* = .221, no interaction between group and age, *F*(2, 98) = 0.144, *p* = .866, and no interaction between period, group, and age, *F*(4.278, 209.625) = 0.991, *p* = .417.

Results of this first analysis allowed us to explore the differences between the contingent and non‐contingent groups in terms of combined arm activity – which was the subject of our hypotheses. However, it is likely that behavioural differences between the two groups may also be observed in the activity of each arm. For this reason, we conducted an analysis comparing the evolution of infants' right and left arm activity by period, group, and age. Note that this analysis was not planned before the experiment. The repeated measures ANOVA showed no main effect of group, *F*(1,98) = 0.288, *p* = .593, no main effect of arm, *F*(1, 98) = 0.023, *p* = .879, no interaction between arm and group, *F*(1, 98) = 0.105, *p* = .747, and no interaction between period, arm, and group, *F*(2.336, 228.918) = 0.812, *p* = .462. Moreover, the ANOVA showed no effect of age, *F*(2, 98) = 1.553, *p* = .221, no interaction between arm, group, and age, *F*(2, 98) = 0.601, *p* = .550, and no interaction between period, arm, group, and age, *F*(4.672, 228.918) = 2.03, *p* = .954. These results suggest that there is no difference in right/left arm activity between the contingent group and the non‐contingent group.

#### Assessment of arm differentiation

**Figure 3:**
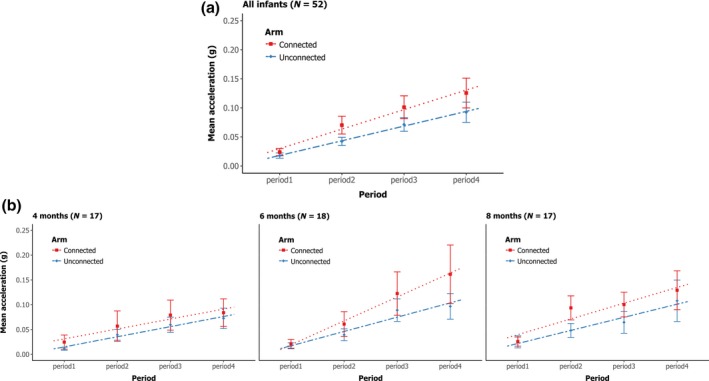
Means and standard errors of the mean of each arm's activity over the four periods of the experiment and the corresponding regression lines for the connected arm (red squares and dotted lines) and the unconnected arm (blue diamonds and dash‐dotted lines). (a) all infants (b) separated by age (4, 6, and 8 months).

In this section, we present only results for the contingent group. We expected that infants who narrowed down the contingency to their connected arm should show higher arm activity or a higher increase over the experiment in arm activity for the connected arm than for the unconnected arm. We also expected that this difference in activity between the connected and the unconnected arm would gradually increase with age (4, 6, and 8 months of age). Figure 3 presents infants’ mean arm activity over the four periods of the experiment for each arm for all infants (Figure 3a) and at each age (Figure 3b).

We see in the top graph with all infants (Figure 3a) that both arm activities increase over the course of the experiment and that the connected arm's activity is globally greater than the unconnected arm's activity. Indeed, the ANOVA shows a significant main effect of period, *F*(1.655, 81.08) = 15.38, *p* « .0001, η^2^p = .358, and a significant main effect of arm, *F*(1, 49) = 5.154, *p* = .028, η^2^p = .095. We did not find an interaction between period and arm, *F*(2.07, 101.428) = 2.146, *p* = .120, suggesting that each arm activity increased equally over time.

In the lower graphs (Figure 3b), we see the same pattern for all age groups. The ANOVA shows no effect of age, *F*(2, 49) = 0.435, *p* = .650, no interaction between arm and age, *F*(2, 49) = 0.185, *p* = .831, and no interaction between period, arm, and age, *F*(4.140, 101.428) = 1.471, *p* = .215. This lack of effect was also confirmed in a supplementary analysis on the rates of increase in the means of the infants’ individual arm activity over the four periods of the experiment.

Moreover, in order to determine whether the difference in activity between the two arms (connected and unconnected) observed in the contingent group was due to exposure to the contingency, we conducted a similar analysis to compare activity between the two arms (right and left) in the non‐contingent group. Note that this analysis was not planned before the experiment. The repeated measures ANOVA showed no significant main effect of arm, *F*(1, 49) = 0.011, *p* = .916, and no interaction between period and arm, *F*(2.139, 104.787) = 0.525, *p* = .605. Moreover, the ANOVA shows no effect of age, *F*(2, 49) = 1.191, *p* = .312, no interaction between arm and age, *F*(2, 49) = 0.493, *p* = .614, and no interaction between period, arm, and age, *F*(4.277, 104.787) = 1.231, *p* = .302. These results suggest that there is no difference in arm activity (right vs. left) in the non‐contingent group, in contrast to the difference in arm activity (connected vs. non‐connected) observed in the contingent group.

## Discussion

The first aim of the present study was to investigate whether 4‐, 6‐, and 8‐month‐old infants would demonstrate sensitivity to a contingency between movements of one of their arms and an audiovisual stimulation. To check for this, we compared the infants’ overall arm activity to the arm activity of a control group that saw an equally salient but non‐contingent audiovisual stimulation. It is important to point out here that we consider the motor response given by an infant when exposed to a sensorimotor contingency as an indicator of the infant's sensitivity to this sensorimotor contingency. We confirmed that infants were sensitive to the contingency. This sensitivity did not manifest itself as a higher overall arm activity in the contingent group but only as a greater increase in arm activity over the course of the experiment in the contingent group compared to the non‐contingent group. This can be explained by supposing that whereas both groups of infants increased their general arousal over the course of the experiment, infants in the contingent group gradually discovered the contingency and so started moving more as compared to the non‐contingent group. Interestingly, contrary to what we expected, we found no evidence that older infants were more sensitive to the contingency than younger ones. The second purpose of our experiment was to assess whether the infants would be able to use their arms in a differentiated manner. We found evidence for arm differentiation when age groups 4, 6, and 8 were taken together: overall, infants moved the connected arm more than the other (unconnected) arm. However, again surprisingly, we had no evidence for progression of this differentiation with age. We shall now discuss these results in more detail.

### Sensitivity to sensorimotor contingencies

Our finding that infants were sensitive to our contingency – suggested by the difference in motor activity observed between the contingent group and the non‐contingent group – is consistent with previous findings (DeCasper & Fifer, 1980; Rovee & Rovee, 1969; Watson, 1972). It is worth noting that our study differs from previous work by the fact that it is the first time sensitivity to contingencies has been demonstrated using wireless accelerometers. Wireless accelerometers are a promising new tool that can be used on infants over a wide range of ages. They provide a convenient measure of motor activity and can be used to establish a variety of types of contingent stimulation (e.g., adding delays between action and feedback, and testing feedback in different sensory modalities).

The result showing no statistical difference in sensitivity to the contingency across age groups is surprising. An explanation might be that the kinds of contingencies that infants are sensitive to change as a function of age (Bahrick & Watson, 1985). Indeed, at 8 months, infants are particularly interested in reaching and grasping, and spend considerable time exploring their environment via the proximal contingencies involved in hand manipulation (see, for instance, Palmer, 1989; Ruff, 1984). However, the contingency used in our study involved no hand manipulation and was distal. This might have prevented 8‐month‐olds from showing more sensitivity to the contingency as compared to 6‐ and 4‐month‐old infants. This might be interesting to test in future studies.

### Assessment of arm differentiation

Our results show evidence that infants are able to move the particular arm that controlled the contingency more than the other arm. This is broadly compatible with other studies showing limb differentiation in infants as early as 3–4 months of age (Angulo‐Kinzler, 2001; Heathcock *et al.*, 2005; Rovee‐Collier *et al.*, 1978; Watanabe *et al.*, 2011; Watanabe & Taga, 2006, 2009). However, the difference between the connected and the unconnected arm did not increase across time during the experiment, neither did it increase across age, contrary to other published studies (Heathcock *et al.*, 2005; Watanabe & Taga, 2006). Presumably, this difference derives from differences in methodologies that we discuss below.

A first difference between our protocol and protocols of other studies using the mobile paradigm concerns the type of contingent feedback involved. In other studies, an infant's arm or leg is attached to the mobile with a ribbon. This provides local tactile stimulation every time the infant moves its connected arm or leg. This was not the case in our experiment, where our wireless technology provided no local tactile feedback to the infant's limb. The presence of co‐located tactile feedback might influence the ability of young infants to narrow down a contingency to a specific limb, and this may account for why the signs of differentiation we observed (i.e., a stable difference in activity between the connected and the unconnected arm) differ from those found in the literature (i.e., an increasing difference between the connected and the unconnected limb across time during the experiment). This hypothesis is supported by the fact that the other existing experiment on limb differentiation using a digital link also revealed divergent results from the rest of the literature, as the authors even failed to show evidence of limb differentiation at 3–4 months of age (Angulo‐Kinzler *et al.*, 2002). Thus, the distinction between co‐located versus distal feedback would be interesting to test in future studies.

A second difference in our protocol compared to others is related to the shorter overall duration of exposure to the contingency (about 4 min in our experiment instead of 6–15 min during one or several sessions in studies using other protocols). This might have allowed infants less opportunity to narrow down the contingency to their connected arm. This hypothesis is supported by the results of Rovee‐Collier *et al. *(1978) in which it is only at the end of the exposure to the contingency (4 days) that all infants showed limb differentiation. Thus, we can suppose that if infants had had more time to explore our contingency, we might possibly have found evidence of limb differentiation comparable to that obtained in the literature.

A last explanation might come from the threshold for triggering the stimulation in our set‐up. Indeed, in our set‐up, even a very small acceleration of the connected arm produced an effect, whereas in the classical mobile paradigm only large flexion‐extension movements of the limb produced an effect (e.g., in Watanabe & Taga, 2006). Thus, in our set‐up the contingent effect could have been produced by any arbitrary body movement provided that it resulted in a small movement of the connected arm. To check for this, we did a qualitative analysis of the videos in order to see whether some infants in the contingent group repetitively adopted some particular specific action other than moving the connected arm and that might have triggered the audiovisual stimulus. We called such alternative triggering behaviour ‘stereotypical behaviour’, because it necessarily involved several repetitions of the same behaviour, whatever it was. The analysis was conducted for all infants, and the coder was not aware of the age (4, 6, or 8 months), the group (contingent or non‐contingent) nor of which arm (if any) was connected. All infants’ repeated actions with a clear anticipatory behaviour towards the audiovisual effect were coded (e.g., the infant starts to kick only when the audiovisual feedback goes off and looks at the screen in anticipation before the smiley moves). We identified five such movements: making large head movements from right to left, moving both arms, kicking, vocalizing, and moving the upper body. This supplementary qualitative analysis suggested that none of the infants in the non‐contingent group presented stereotypical behaviour but some infants in the contingent group indeed used an alternative action while exploring the sensorimotor contingency. These behaviours were mainly observed in the older infants (*N* = 7 at 6 months and *N* = 7 at 8 months) and less in the younger infants (*N* = 2 at 4 months). Though debatable, there is a way of interpreting this result as being compatible with the hypothesis that body know‐how develops from global to local. It could be that even though older infants are not able to correctly localize the connected body part, at least they are systematically and repetitively moving a *specific* body part, contrary to younger infants that indiscriminately move their whole bodies. Moreover, this supplementary qualitative analysis raises a methodological point regarding the threshold setting. On the one hand, using a very low threshold seems to facilitate contingency detection in that it gives the infant the opportunity to discover the contingency by chance. On the other hand, a high threshold would facilitate the production of a specific local response (Watson, 1972; Zwicker, Moore & Povinelli, 2011). The question of which threshold to choose remains open.

### Conclusion

Our paper provides new insights into the development of body knowledge during early infancy. Based on the hypothesis that body know‐how – the ability to sense and use the body parts in an organized and differentiated manner – develops from global to local in the first month of life, our aim was to address the lack of studies on limb differentiation in infants older than 4 months. We demonstrated that 4‐ to 8‐month‐old infants seem able to use their arms in a differentiated manner when movements of only one of their arms generate a contingent audiovisual feedback. However, we were not able to show a developmental trend in arm differentiation between 4 and 8 months of age. In future work, it will be interesting first to test younger infants so as to determine at what moment the global‐to‐local transition in body know‐how occurs. Second, it will be interesting to test how the kinds of contingencies (e.g., analogue vs. digital, local vs. distal, or haptic vs. non‐haptic) that infants are best at detecting and narrowing down depend on the infants’ age and/or motor abilities. The wireless technology using Bluetooth accelerometers developed in this study appears to be a good tool to create such adaptable contingencies. To conclude, further work is needed to better understand how body know‐how develops and is fine‐tuned over the first year of life so as to provide the properly differentiated sense of the body essential for interacting with the physical and social world.

## Conflicts of interest

All authors declare no conflict of interest.
